# Portable Raman leaf-clip sensor for rapid detection of plant stress

**DOI:** 10.1038/s41598-020-76485-5

**Published:** 2020-11-19

**Authors:** Shilpi Gupta, Chung Hao Huang, Gajendra Pratap Singh, Bong Soo Park, Nam-Hai Chua, Rajeev J. Ram

**Affiliations:** 1 Disruptive & Sustainable Technologies for Agricultural Precision, Singapore-MIT Alliance for Research and Technology, 1 Create Way, #03-06/07/8 Research Wing, Singapore, 138602 Singapore; 2 Temasek Life Science Laboratory, 1 Research Link, National University of Singapore, Singapore, 117604 Singapore; 3 Research Laboratory of Electronics, Massachusetts Institute of Technology, 77 Massachusetts Avenue, 36-491, Cambridge, MA 02139 USA

**Keywords:** Optics and photonics, Plant stress responses

## Abstract

Precision agriculture requires new technologies for rapid diagnosis of plant stresses, such as nutrient deficiency and drought, before the onset of visible symptoms and subsequent yield loss. Here, we demonstrate a portable Raman probe that clips around a leaf for rapid, in vivo spectral analysis of plant metabolites including carotenoids and nitrates. We use the leaf-clip Raman sensor for early diagnosis of nitrogen deficiency of the model plant *Arabidopsis thaliana* as well as two important vegetable crops, Pak Choi (*Brassica rapa chinensis*) and Choy Sum (*Brassica rapa var. parachinensis*)*. *In vivo measurements using the portable leaf-clip Raman sensor under full-light growth conditions were consistent with those obtained with a benchtop Raman spectrometer measurements on leaf-sections under laboratory conditions. The portable leaf-clip Raman sensor offers farmers and plant scientists a new precision agriculture tool for early diagnosis and real-time monitoring of plant stresses in field conditions.

## Introduction

Motivated by food insecurity arising from a growing global population^1–3^ and climate change^4–6^, Precision Agriculture has been proposed as a strategy to increase agriculture productivity while enhancing sustainability. One important objective of Precision Agriculture is to determine the state of plant health before symptomatic manifestation and yield loss arising from abiotic or biotic stresses, i.e. to distinguish the stressed phenotype from the unstressed phenotype. However, characterizing plant phenotypes is costly and time consuming and presents a bottleneck in crop management as well as in the screening of progeny in crop breeding programs^7^. Technologies for rapid stress phenotyping can contribute to enhanced productivity of major food crops around the world^8^.

To address this need, optical techniques have been explored extensively for agricultural phenotyping. Optical imaging is used to measure plant morphology and structure (height, size, leaf area, etc.)^9,10^. Reflectance spectroscopy has been used to assess chemical constituents (e.g. chlorophyll and carotenoids) of plant leaves rapidly and non-destructively^11,12^. These nondestructive measurements allow repeated measurements for the quantification of a plant’s dynamic response including growth and stress response^13,14^. However, changes in the reflectance or transmittance are similar for several abiotic stresses^15–17^ as well as general stress response^18–21^. Stress markers associated with changes in spectral reflectance also take time to accumulate; moreover, these markers may not allow for early stress detection to mitigate yield loss. Raman spectroscopy offers the possibility of a specific and early diagnostic tool^18,22–29^.

In recent years, there has been growing interest in Raman spectroscopy as a high-content phenotyping tool for Precision Agriculture. Handheld Raman spectrometers have been used to monitor plant health and development, early diagnosis of disease, and biotic and abiotic stresses in plants^18,22–29^. In the reported literature for plant Raman spectroscopy^18,28–31^, either a leaf sample was collected from plants and stored until spectrum acquisition^20,26–28^ or whole plants were positioned around a spectrometer and leaves were placed on a sample holder without physical detachment from the plant^18,29,30^. Here, we demonstrate a leaf-clip based Raman probe that allows for rapid, reproducible in vivo measurements on plants growing in plant rooms and farms.

Raman spectroscopy measures the shift in the energy of a scattered photon resulting from the interaction of the photon with the vibrational or rotational states associated with the covalent bonds in the constituent molecules^31,32^. A Raman spectrum can be treated as a chemical fingerprint for different compounds as each peak in the Raman spectrum corresponds to a particular Raman active vibration of a molecule. However, this inelastic Raman scattering process is weak when compared to Rayleigh scattering or with direct absorption of photons. Leaf-clip sensors have previously only been used to assess leaf absorption (transmittance) or fluorescence. The leaf-clip format for optical sensing was first introduced by Inada^33,39^ and transmission based leaf-clip sensors have been commercialized by several vendors^34,35^. These leaf-clip sensors for chlorophyll are affected by leaf anatomy, cuticle reflectance, leaf veins, and the flatness of leaves^36,37^. Variability in the transmission and absorption of light due to leaf heterogeneity requires careful calibration for each plant species.

### Instrumentation for portable Raman leaf-clip

The leaf-clip Raman sensor demonstrated here uses a Raman fiber probe connected to a portable Raman instrument using an 830 nm excitation laser. The leaf-clip simultaneously: (1) maintains the probe-to-leaf sample distance, (2) gently holds the leaf so as to suppress relative beam displacement over the leaf surface, (3) blocks ambient light, (4) blocks the transmitted laser radiation rendering the device eye-safe, and (5) provides a comfortable handhold. As described below the multiplicity of analytes observable in the Raman spectra allows us to establish internal references that make Raman-based diagnostics less sensitive to variations in transmission and absorption as previously described leaf-clip sensors.

**Figure 1:**
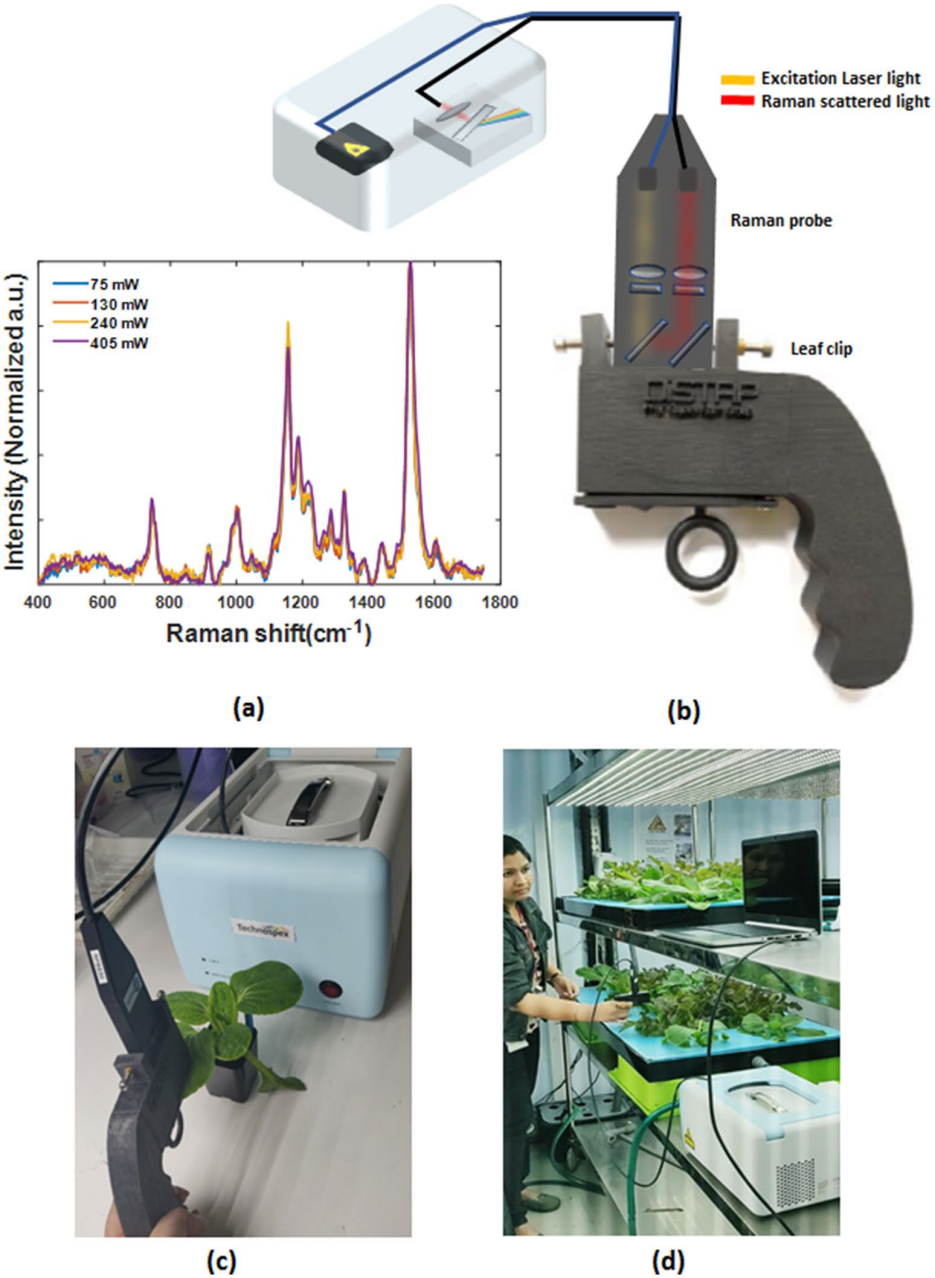
Portable leaf-clip Raman sensor and its use on plants. (**a**) Raman spectra of Arabidopsis under different laser power 75 mW (minimum), 130 mW (used in experiments), 240 mW and 405 mW (maximum). (**b**) Schematic of the portable Raman spectrometer, (**c**) Raman Leaf-Clip sensor clipped with plant, (**d**) the leaf-clip Raman sensor used for analysis of early nitrogen deficiency in leafy vegetables.

The leaf-clip Raman sensor consists of a 3D printed clip that is built around a fiber-based Raman probe assembly. An anodized aluminium Raman probe (InPhotonics) (4.2″ × 1.5″ × 0.5″) with 1.5″ long stainless-steel tip is used for sample illumination and light collection optics. The Raman probe^38^ incorporates a minimum number of optical components in a small, robust probe-head for optimum performance and experimental versatility. Infrared laser excitation light at 830 nm is supplied through a 105 µm core fiber. The probe assembly includes thin film filters to remove amplified spontaneous emission from the excitation source. This excitation light is focused onto the sample through a focusing lens and window with a useful working distance of 7.5 mm. Back-scattered Raman light is collected by the same lens and transmitted through an excitation blocking filter. Figure 1a shows the Raman spectra of the plant leaf sample illuminated by 830 nm laser with different laser power. Varying the laser power from 75 mW (minimum) to 405  mW (maximum) shows no significant difference in the acquired Raman spectra. Here, typical operating conditions utilize 130 mW of 830 nm laser excitation at the sample.

The leaf-clip interface was designed to quickly and reproducibly place the leaf at a fixed distance from the collection lens in the fiber probe. Stereolithographic 3D printing of methacrylate photopolymers was used to create a holder for the fiber probe and the leaf (Fig. 1c,d). The probe is held firmly in place with set-screws and the leaf is held in place by a 3D printed clip with embedded magnets. Opposing magnets are used to hold the leaf steady without otherwise damaging the leaf tissue. The clip also blocks leakage of daylight into the Raman probe and incorporates a tilted surface to block the transmitted laser light for eye-safe operation and suppress back-reflection into the fiber probe.

The laser (Innovative Photonic Systems USA, 830 nm excitation) and spectrometer (Avantes HSC Symmetrical Czerny-Turner, 100 mm focal length, NA: 0.13 optical bench and a TE cooled 1024 × 58 back thinned CCD detector) were integrated into a single, portable instrument box with software controls by TechnoSpex Pte Ltd (Fig. 1b). The spectral span of the instrument was 100 cm^−1^ to 2000 cm^−1^ and the spectral resolution was 10 cm^−1^.

### Reference benchtop Raman instrument

Reference Raman measurements were performed in the laboratory and spectra were acquired using Kymera 328i spectrograph (Andor, UK) employing a 600 g mm^−1^ optical grating and 830 nm excitation. We measured the Raman spectra of leaf sections placed on a 100 µm thick fused silica slide, from two locations per leaf (one on each side of the midvein) from the entire cross-section of a leaf^39^. In Ref.^39^, nitrate measurements using this benchtop Raman instrument were validated against chemical analysis of nitrate^40^ for several plant species. Further validation was acquired by measurements of wild-type *Arabidopsis thaliana* as well as mutants affected in metabolic pathways associated with nitrogen uptake. Reference^39^ also demonstrates that time-series Raman spectra provide early diagnosis of nitrogen deficiency and can be used to guide crop management.

### Spectral analysis

The designed Raman leaf-clip sensor is demonstrated for in vivo and in situ acquisition of Raman spectra under growing conditions for a wide range of vegetable plants (Kailan, Lettuce, Choy Sum, Pak Choi, and Spinach) as well as the model plant *Arabidopsis thaliana*. For each sample, 5 spectra were collected with an integration time of 10 s per sample spot. Cosmic ray events were identified in the 10 s spectra and removed. After cosmic ray removal, the individual 10 s spectra were smoothed across wavelength using the Savitzky-Golay filter function (MATLAB Inc., USA). Savitzky-Golay filter is used to smooth spectra in order to reduce the impact of noise on statistical classification^41,42^. A representative sample spectrum was created by taking the mean value of the five filtered and smoothed spectra at each wavelength. The sample spectrum resulting from this processing contained Raman and fluorescence signal. To generate the Raman spectrum presented in the results section any residual fluorescence was removed by performing a positive residual style polynomial subtraction as described in reference^42^. All the processing was done in the MATLAB programming language. The Raman spectrometer provides a built-in frequency reference as attenuated 830 nm laser light is observed in the Raman spectra. Calibration of the Raman shift was also checked using polystyrene with a well-known Raman spectrum^43^.

**Figure 2:**
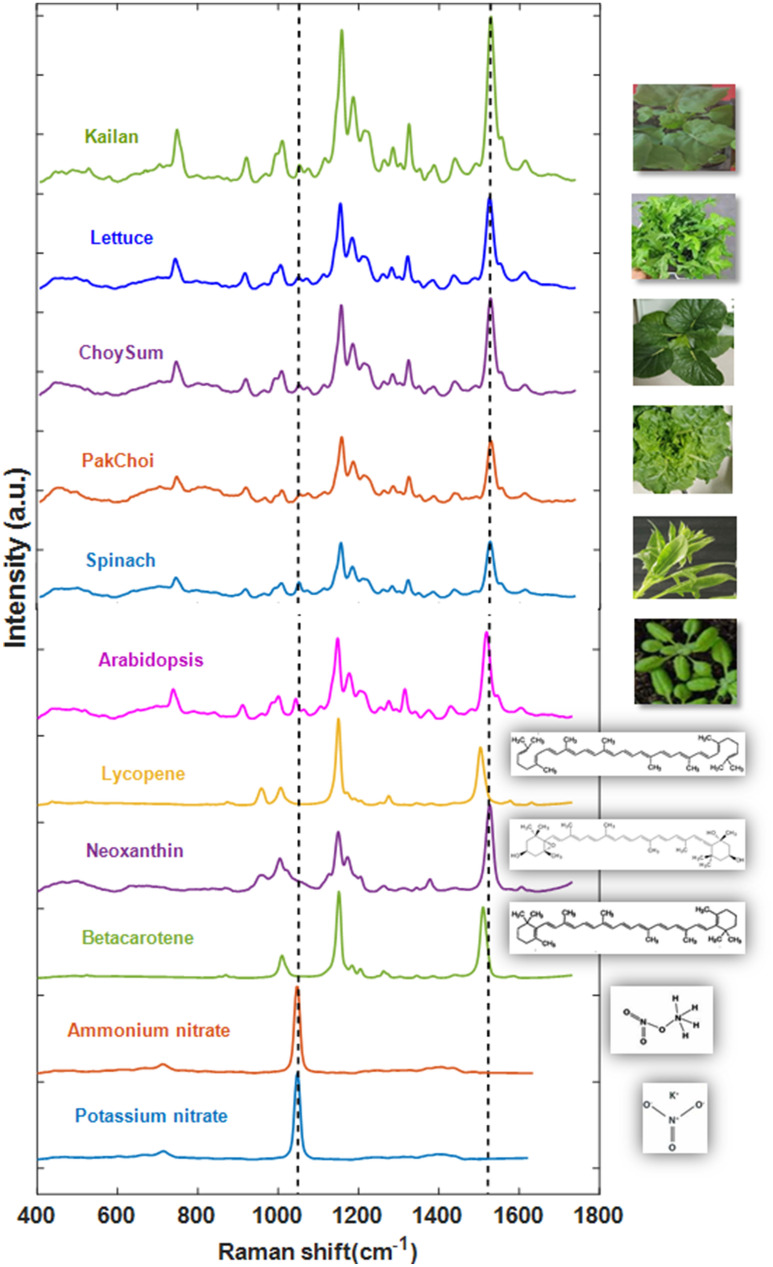
Raman spectroscopic analysis of leafy vegetables (Kailan, Lettuce, Choy Sum, Pak Choi, Spinach), Arabidopsis and Phytonutrients (Lycopene, Neoxanthin and Beta Carotene) and macronutrient (Nitrate peak by 100 mM NH_4_NO_3_ and KNO_3_) of plants by standard pure chemicals.

A preliminary experiment was performed for phytonutrients (carotenoids) and macronutrient (nitrogen) standards, as well as *Arabidopsis thaliana* plants and 5 different leafy vegetable plants as shown in Fig. 2. Based on our Raman spectrum of carotenoid chemical standards, we identified the 1520 cm^−1^, 1150 cm^−1^ and 1004 cm^−1^ peaks to be present in all tested carotenoids (Fig. 2). The observed Raman peak at 1520 cm^−1^ is due to a C=C stretching vibration (ν_1_), the 1150 cm^−1^ peak is a C–C stretching vibration (ν_2_), and the weak peak at 1004 cm^−1^ is due to C-CH_3_ stretching (ν_3_)^44^. Furthermore, the measured Raman spectrum of potassium nitrate (KNO_3_) and ammonium nitrate (NH_4_NO_3_) showed a peak at 1046 cm^−1^ indicating that this Raman shift (associated with the symmetrical stretching of nitrate) is indeed the nitrate peak^39^ (Fig. 2).

For the Raman analysis, 3-week old, *Arabidopsis thaliana*, Lettuce (*Lactuca sativa*), Pak Choi (*Brassica rapa chinensis*), and Choy Sum (*Brassica rapa var. parachinensis*) plant, were grown in hydroponic media and Kailan (*Brassica oleracea var. alboglabra*) and Spinach (*Spinacia oleracea*) grown in soil were chosen, which have different leaf shapes, sizes, and textures. The *Arabidopsis thaliana* leaf was small (1.5–4 cm), smooth and covered with unicellular hairs, whereas Pak Choi, Choy Sum and Lettuce leaf were bigger, and surfaces were semi savoyed to heavily savoyed (crinkled). Similarly, the Spinach leaf was triangular or ovate in shape with a very smooth surface and Kailan leaf was thick, oblong, and glossy (coated with wax). The Raman Leaf-Clip Sensor was able to acquire Raman spectra from all the different types of plant leaves with reproducible results. The Raman spectra of plants leaves shows several common Raman peaks. The vibrational bands and their assignments for *Arabidopsis thaliana* and leafy vegetable plants are listed in Table 1.

**Table 1:** Vibrational bands and their assignments for *Arabidopsis thaliana*, and leafy vegetable plants.

Band (Raman peak cm^−1^)	Vibrational mode	Assignment
1604	ν(C–C) aromatic ring + σ(CH)	Lignin^20,27^
1520	–C=C– (in-plane)	Carotenoid^20,27,43^
1320	δCH2 bending	Cellulose, Lignin^20,27^
1150	C–C stretching; v(C–O–C),	Carotenoid^27,43^
1046	NO_3_ stretching	Nitrate^38^
1004	ν3 (C–CH3 stretching)	Carotenoids^20,43^
1003	ν3 (C–CH3 stretching) phenylalanine ring stretching mode	Phenylalanine^20,43^
747	γ(C–O–H) of COOH	Pectin^20,27^

### Plant materials and plant growth

*Arabidopsis thaliana* WT (Col-0), Pak Choi (*Brassica rapa chinensis*) and Choy Sum (*Brassica rapa var. parachinensis*) were grown in Temasek Life Science Laboratory. Seeds were germinated on 0.8% agar media containing Murashige and Skoog (MS) salts, 0.5 g L^−1^ MES and 10 g L^−1^ sucrose. Arabidopsis and vegetables were grown at 22 °C with 60% relative humidity in long-day conditions (16 h light/8 h dark) under white light at 100 μmol m^−2^ s^−1^ in a growth chamber. Plants were grown in either + N or −N medium by modified Hoagland’s solution containing 2 mM CaCl_2_ and 3 mM KCl (pH 5.8) instead of 2 mM Ca(NO_3_)_2_ and 3 mM KNO_3_ (pH 5.8).

In order to demonstrate the broad applicability of the Raman leaf-clip, additional experiments on soil based plants grown under drought and heat stress conditions were performed. Choy Sum (*Brassica rapa var. parachinensis*) were grown in the DiSTAP Laboratory. Seeds were germinated on 0.8% agar media at 25 °C in a growth chamber. After germination Choy Sum vegetables plant were grown in soil at 20 °C with 60% relative humidity in long-day conditions (16 h light/8 h dark) under white light at 100 μmol m^−2^ s^−1^ in a plant room. Three week old Choy Sum plants grown in the control condition were kept in drought and high-temperature stress for 3 days. These plants were used to acquire Raman spectra.

### Monitoring stress response

The Raman Leaf-Clip Sensor was used for early detection of nitrogen status in plants. Nitrogen (N) plays a key role in plant growth and the entire life cycle of a plant. It is the main plant macronutrient needed for chlorophyll production and other plant cell components e.g. amino acid, nucleic acids, and protein biosynthesis^45^. Nitrogen limitation promotes premature leaf senescence and a reduction in chlorophyll lowering yield and biomass. If the available nitrogen exceeds the plant’s nutritional needs then the excess is eliminated by runoff and infiltration into the water table leading to pollution of aquatic ecosystems. Thus, the optimization of nitrogen deficiency has become the object of intense importance for plant health as well as its environmental and economic impact^46,47^.

We choose the model plant *Arabidopsis thaliana* where metabolic pathways are well studied and two leafy vegetable plants belonging to the *Brassicacea* family: Pak Choi (*Brassica rapa chinensis*) and Choy Sum (*Brassica rapa var. parachinensis*) for the experiment. Three-week-old plants were grown under sufficient (+ N; complete) or nitrogen-deficient (−N) hydroponic media. After 5 days, leaf yellowing which reflects senescence induced by nitrogen starvation was not yet visible in plants grown under nitrogen-deficient conditions. However, despite the similarity in visible plant phenotype and chlorophyll content, chemical analysis showed the nitrate content of −N plants was decreased, compared to + N plants. These plants were used to acquire Raman spectra from portable system as well as benchtop system.

### Statement of consent

The informed consent was obtained from the participant researcher for publication of photograph in the article (Fig. 1d).

## Results and discussion

We first sought to test the fidelity of the Raman spectral measurements on the heterogeneous surface of the leaf. Raman measurements with the leaf-clip probe are not guided by machine or human vision. Raman spectra were acquired from *Arabidopsis thaliana* grown under sufficient (+N) and deficient (−N) condition of Nitrogen (N) as described above. For each growth condition, 5 biological replicates were tested (a total of 10 plants). Two locations (left and right of centre) on a single leaf were measured on each plant. At each location, five Raman spectra were acquired. In the Supplementary Information, we demonstrate that a nitrate Raman measurement from two locations gives a comparable estimate of the nitrate peak ratio as a whole leaf map using 30 locations. In vivo and in situ measurements with the leaf-clip Raman sensor and portable instrument were compared to experiments performed against the benchtop system on 3 mm diameter leaf sections placed on a fused silica cover slip^39^.

**Figure 3:**
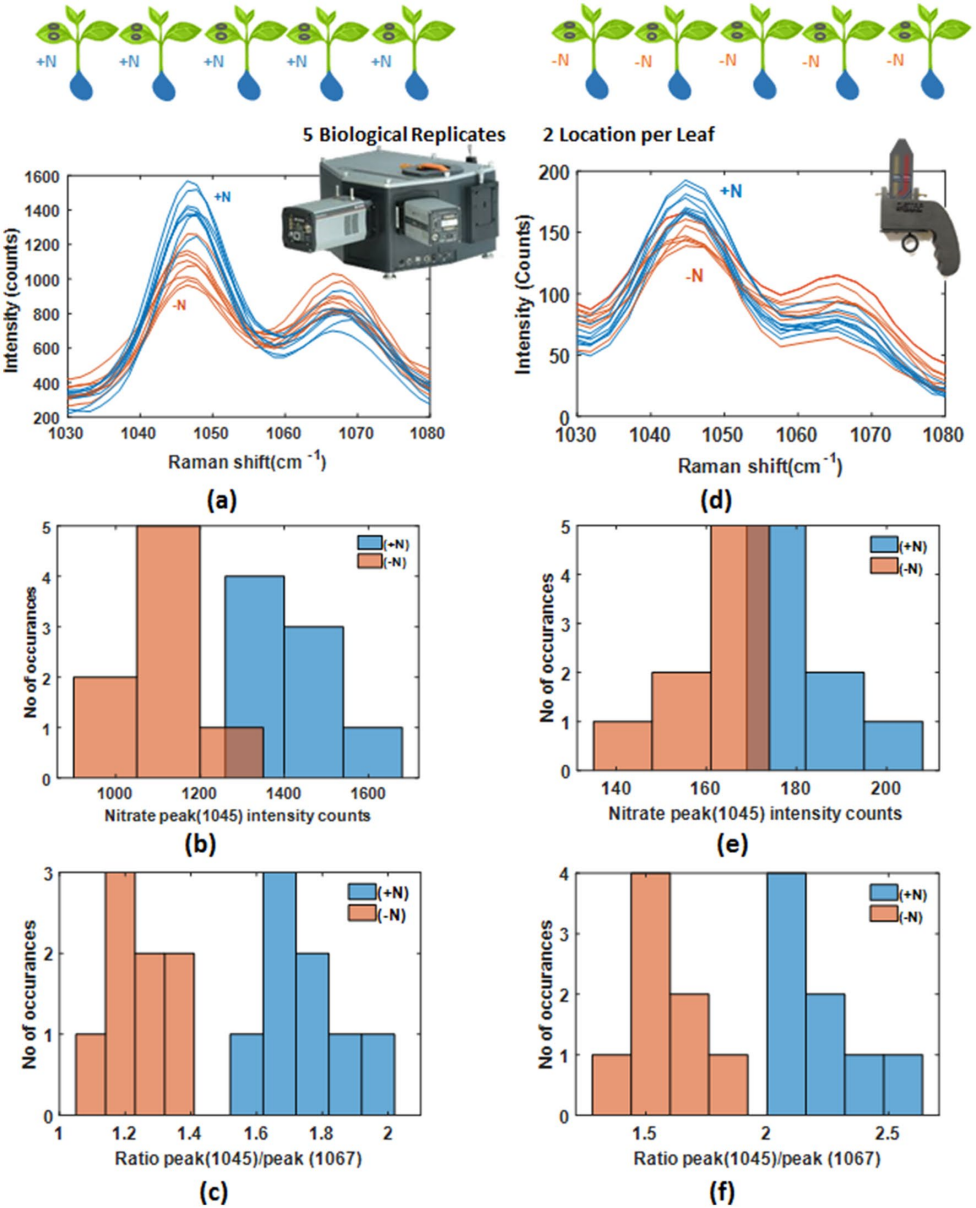
Reproducibility of Raman spectra under Nitrogen sufficient (+ N) or deficient (− N) condition in Arabidopsis. (**a**) Raman spectra of 8 samples each from + N and − N Arabidopsis plants were measured by Benchtop Raman spectrometer (Region of Raman spectra between 1030 and 1080 cm^−1^ is shown) (**b**) Histogram of 1045 cm^−1^ Nitrate Raman peak of 8 samples each from + N and − N Arabidopsis plants by Benchtop Raman spectrometer, (**c**) Histogram of ratio of 1045 cm^−1^ Nitrate Raman peak to 1067 cm^−1^ adjacent peak (1045 cm^−1^/1067 cm^−1^) of 8 samples each from + N and − N Arabidopsis plants by Benchtop Raman spectrometer, (**d**) Raman spectra of 8 samples each from + N and − N Arabidopsis plants were measured by Portable Raman spectrometer (Region of Raman spectra between 1030 and 1080 cm^−1^ is shown) (**e**) Histogram of 1045 cm^−1^ Nitrate Raman peak of 8 samples each from + N and − N Arabidopsis plants by Portable Raman spectrometer, (**f**) Histogram of ratio of 1045 cm^−1^ Nitrate Raman peak to 1067 cm^−1^ adjacent peak (1045 cm^−1^/1067 cm^−1^) of 8 samples each from + N and − N Arabidopsis plants by Portable Raman spectrometer.

The region of Raman spectra between 1030 and 1080 cm^−1^ as shown in Fig. 3a,d indicates that the 1045 cm^−1^ peak with sufficient (+ N) and deficient (− N) condition are clearly distinguishable. Histograms of the 1045 cm^−1^ nitrate Raman peak intensity for 8 spectra from + N and − N Arabidopsis plants was plotted for both benchtop leaf sections and portable, in vivo leaf-clip Raman instruments shown in Fig. 3b, e, respectively. The variability in the intensity of the Raman peak is likely due to the excitation overlapping leaf veins or microscopic variations in the surface orientation and reflection.

We can improve the classification of Raman spectra from sufficient (+ N) and deficient (− N) conditions by using the 1067 cm^−1^ peak as an internal reference. Histograms of the ratio of 1045 cm^−1^ to the 1067 cm^−1^ adjacent peak (1045 cm^−1^/1067 cm^−1^) of Arabidopsis plants are presented in Fig. 3c, for the benchtop Raman system on leaf sections, and in Fig. 3f for the leaf-clip Raman sensor on in vivo plants. This internal referencing results in a smaller variation across biological replicates and leaf locations and gives a clear separation in the Raman intensity ratio for sufficient and deficient nutrient conditions (Supplementary Table 1).

To calculate the signal to noise ratio (SNR) of both the benchtop and portable system, 30 spectra were collected with an integration time of 10 s at same location from two biological replicates. The calculated value of SNR of the nitrate peak (1045 cm^−1^) of Choy Sum plants is SNR = 5.15 and 5.31 for benchtop Raman system and SNR = 2.51 and 3.31 for the portable system. Using the adjacent peak as an internal reference improved the relative performance of the portable leaf-clip system, the SNR of the ratio of the nitrate peak 1045 cm^−1^ to the 1067 cm^−1^ adjacent peak (1045 cm^−1^/1067 cm^−1^) of Choy Sum plants is SNR = 6.28 and 6.38 for the benchtop Raman system and SNR = 5.05 and 5.06 for the portable system. Despite the smaller light collection of the Raman leaf clip and the utilization of miniature components in the portable instrument, the SNR for the peak ratios are comparable between the in vivo measurements using the portable leaf-clip sensor and leaf samples in the benchtop systems.

**Figure 4:**
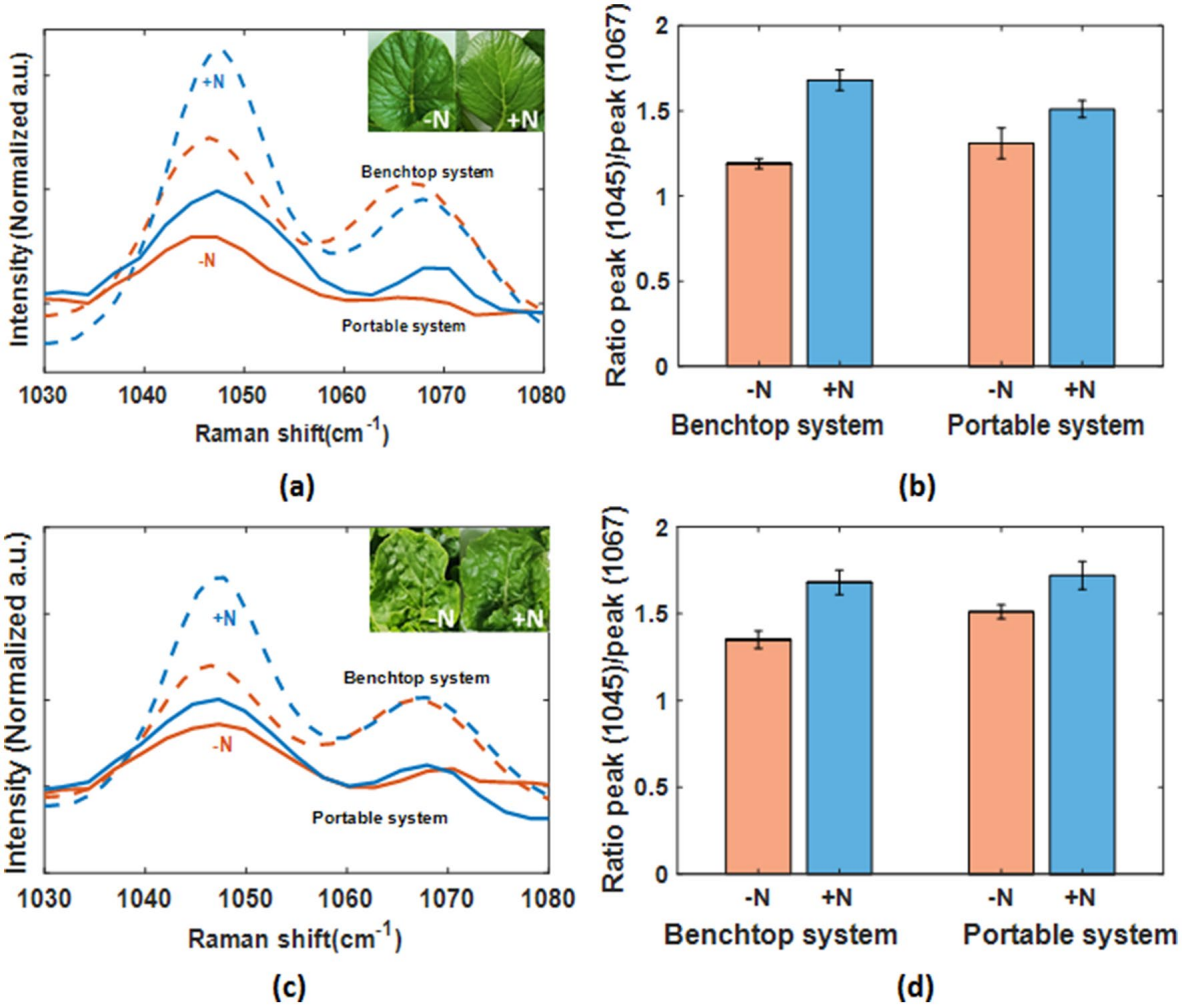
Comparison of Raman spectra under Nitrogen sufficient (+ N) or deficient (− N) condition in leafy vegetables (Choy Sum and Pak Choi), acquired by Benchtop Raman spectrometer and Portable Raman spectrometer. (**a**) Raman spectra of + N and − N Choy Sum plants by Benchtop Raman spectrometer and Portable Raman spectrometer (Data are mean values, of 3 biologically independent experiments shown in region of Raman spectra between 1030 and 1080 cm^−1^), (**b**) comparison of ratio of 1045 cm^−1^ Nitrate Raman peak to 1067 cm^−1^ adjacent peak (1045 cm^−1^/1067 cm^−1^) in + N and − N Choy Sum plants by Benchtop Raman spectrometer and Portable Raman spectrometer, (**c**) Raman spectra of + N and − N Pak Choi plants by Benchtop Raman spectrometer and Portable Raman spectrometer (Data are mean values, of 3 biologically independent experiments shown in region of Raman spectra between 1030 and 1080 cm^−1^), (**d**) comparison of ratio of 1045 cm^−1^ Nitrate Raman peak to 1067 cm^−1^ adjacent peak (1045 cm^−1^/1067 cm^−1^) in + N and − N Pak Choi plants by Benchtop Raman spectrometer and Portable Raman spectrometer.

These results can be extended to vegetable crops. Raman spectra were acquired from 3 biological replicates with 2 location at each leaf of same age for Pak Choi and Choy Sum (Fig. 4). For each leaf location, 5 spectra were collected with an integration time of 10 s per spot. Every plant spectrum is a mean of 10 spectra. The spectra obtained from + N plants and − N plants showed consistent decreases of nitrate peak intensity from + N plants to − N plants. Here, we have shown that Raman spectroscopy can be used to establish the state of plant health in a non-invasive manner by using the 1045 cm^−1^ Raman peak as a specific signature of nitrogen status in Arabidopsis and vegetable crops.

**Figure 5:**
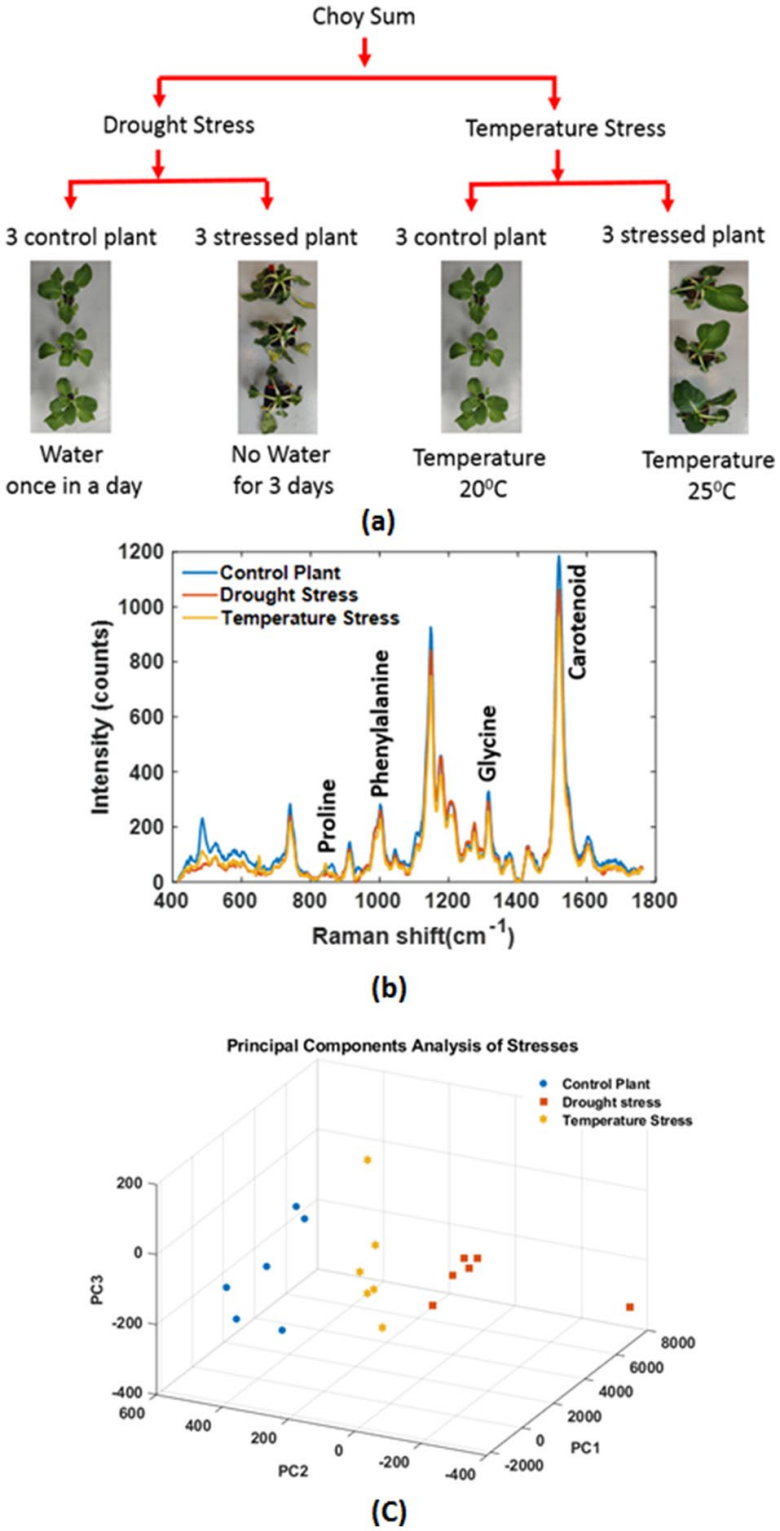
The leaf-clip Raman sensor used for analysis of early diagnosis of drought and high temperature stress in Choy Sum plant. (**a**) Schematic of the experimental plan, (**b**) Raman spectra of control plant and stressed plant (**c**) Principal component analysis method to distinguish the control and different stress plant.

To illustrate the applicability of the Raman Leaf-Clip Sensor to the diagnosis of other stress conditions, experiments were performed on plants under drought and temperature stress conditions. Figure 5a shows the schematic of the experimental plan. Figure 5b shows the Raman spectra of control plant and stressed plant which indicated the variation of secondary metabolites of plants under stressed condition as early as 3 days. Principal component analysis clearly distinguishes the control and plants experiencing the two stress conditions as shown in Fig. 5c.

## Conclusion

Rapid quantification of the stress phenotype associated with nutrient deficiency has been demonstrated using a portable leaf-clip Raman sensor. We have established the reproducibility of the leaf-clip based measurements and demonstrated the diagnosis of nitrogen deficiency for the model plant *Arabidopsis thaliana* as well as two important vegetable crops Pak Choi (*Brassica rapa chinensis*) and Choy Sum (*Brassica rapa var. parachinensis*). Spectra collected in vivo and in situ (under full light growing conditions) using the leaf-clip sensor are shown to be comparable to those from leaf sections measured using a benchtop Raman spectrometer.

While we have focused on the early and specific diagnosis of nitrogen deficiency using the leaf-clip sensor, there are peaks from other metabolites that are also clearly observed in Kailan, Lettuce, Choy Sum, Pak Choi, and Spinach. We expect that the carotenoid and anthocyanin Raman spectra can be used to measure a wider range of plant stress phenotypes—from drought, to heat and cold stress, to saline stress, and light stress^18^. Indeed, preliminary data for drought and heat stress have been presented. The simplicity and speed of these leaf-clip Raman probes makes them suitable for field use by farmers as well as laboratory use by researchers for rapid screening of cultivars for enhanced stress-tolerance.

## Supplementary information

Supplementary Figure 1.

Supplementary Table 1.
