# Supplementary material for: Portable Raman leaf-clip sensor for rapid detection of plant stress
Source: Sci Rep. 2020 Nov 19;10:20206. doi: 10.1038/s41598-020-76485-5 (PMC7677326; doi:10.1038/s41598-020-76485-5)

**Portable Raman Leaf-Clip Sensor for Rapid Detection of Plant Stress**

Shilpi Gupta^1,+^, Chung Hao Huang^2,+^, Gajendra Pratap Singh^1^, Bong Soo Park^2^, Nam-Hai Chua^1,2,*^, Rajeev J. Ram^1,3,*^

**Supplementary Figure 1- Leaf location dependent Raman spectra variability**

(a) 30 different location indication on leaf surface, (b) Histogram of 225 sets from 2 location on the leaf at both side of midvein.


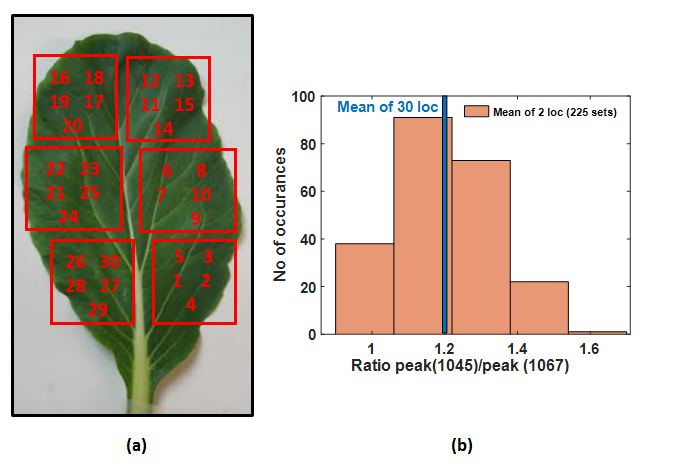

Supplement: Supplementary file 1 — Supplementary Figure 1. [file 41598_2020_76485_MOESM1_ESM.docx]
