# Supplementary material for: Portable Raman leaf-clip sensor for rapid detection of plant stress
Source: Sci Rep. 2020 Nov 19;10:20206. doi: 10.1038/s41598-020-76485-5 (PMC7677326; doi:10.1038/s41598-020-76485-5)
Supplement: Supplementary file 2 — Supplementary Table 1. [file 41598_2020_76485_MOESM2_ESM.docx]

**Portable Raman Leaf-Clip Sensor for Rapid Detection of Plant Stress**

Shilpi Gupta^1,+^, Chung Hao Huang^2,+^, Gajendra Pratap Singh^1^, Bong Soo Park^2^, Nam-Hai Chua^1,2,*^, Rajeev J. Ram^1,3,*^

**Supplementary Table 1- P-value data for Nitrate peak intensity and Ratio of Nitrate peak to adjacent peak intensity (Fig. 3 and Fig. 4)**

| **Plants** | **System** | **Nitrate peak (1045 cm-1)** | **Ratio of Nitrate peak to adjacent peak (1045 cm-1/ 1067 cm-1)** |
| --- | --- | --- | --- |
|  |  |  |  |
| ***Arabidopsis thaliana WT (Col-0)*** | Benchtop system | 2.9298E-04 | 6.0380E-05 |
|  | Portable system | 6.7060E-04 | 1.4479E-04 |
| **Pak Choi (*Brassica rapa chinensis*)** | Benchtop system | 2.4132E-03 | 8.3276E-05 |
|  | Portable system | 8.9998E-03 | 3.5827E-04 |
| **Choy Sum (*Brassica rapa var. parachinensis*)** | Benchtop system | 1.4452E-03 | 7.9678E-05 |
|  | Portable system | 5.5048E-03 | 1.1315E-04 |

Table above lists P values of nitrogen-deprived growth medium (-N) relative to Full medium (+N) as obtained from Student’s t-test analysis (n=5 to 8)
